# Immune checkpoint inhibitor outcomes and prognostic factors in gynecologic tract melanoma: a single-center analysis

**DOI:** 10.3389/fimmu.2025.1542293

**Published:** 2025-04-15

**Authors:** Jianzhang Wang, Kemin Li, Rui Li, Jing Zeng, Rutie Yin

**Affiliations:** ^1^ West China School of Medicine, Sichuan University, Chengdu, Sichuan, China; ^2^ Department of Obstetrics and Gynecology, West China Second University Hospital, Sichuan University, Chengdu, Sichuan, China; ^3^ Key Laboratory of Birth Defects and Related Diseases of Women and Children, West China Second University Hospital, Sichuan University, Chengdu, Sichuan, China

**Keywords:** melanoma, gynecological, immunotherapy, immune checkpoint inhibitors, prognostic factors

## Abstract

**Introduction:**

Gynecologic tract melanoma (GTM) is a rare and aggressive malignancy with limited treatment options and poor prognosis. This study aims to evaluate the outcomes of immune checkpoint inhibitors (ICIs) in patients with GTM and identify prognostic factors influencing survival.

**Methods:**

A retrospective analysis was conducted on 45 patients diagnosed with GTM at West China Second University Hospital from January 2019 to September 2024. Data on demographics, clinical characteristics, treatments, and outcomes were collected. Progression-free survival (PFS) and overall survival (OS) were analyzed using Kaplan-Meier curves and Cox proportional-hazards models.

**Results:**

Among 45 patients, 24 had vaginal melanoma, 18 had vulvar melanoma, and 3 had cervical melanoma. ICIs were administered to 21 patients, but no significant survival benefit was observed. The 1-, 3-, and 5-year survival rates were 87%, 63%, and 31%, respectively. Univariate analysis revealed that patients with a family history of cancer (FHC) and those with lactate dehydrogenase (LDH) levels ≤230 had better PFS. Additionally, FHC, American Joint Committee on Cancer (AJCC) stage I-II, absence of pelvic lymph node metastasis, and LDH levels ≤230 were associated with improved OS. However, in multivariate analysis, only LDH was significantly associated with OS.

**Conclusion:**

This single-center study suggests that ICIs have limited efficacy in treating GTM, emphasizing the need for further investigation through larger, multicenter clinical trials. Prognostic factors such as FHC, AJCC stage, lymph node involvement, and LDH levels may aid in risk stratification and personalized treatment planning. However, due to the nature of this study, external cohorts are still needed for validation.

## 1 Introduction

Gynecologic tract melanoma (GTM) is a rare and highly aggressive form of cancer, accounting for only 3% to 7% of all mucosal melanomas (MM) (1). GTM primarily includes melanomas originating in the vulva, vagina, and cervix, all of which are associated with poor prognoses (2–4). Approximately 18-40% of MM arise in the vulvar region (5). Epidemiological data indicate that the annual incidence of vulvar malignant melanoma is around 0.136 cases per 100,000 people, with 5-year survival rates ranging from 10% to 63% (6). Vaginal malignant melanoma is even rarer, with an incidence of 0.046 cases per 100,000 and a 5-year survival rate of about 15% (7). Cervical malignant melanoma is the rarest subtype, with only 149 reported cases worldwide and a 5-year survival rate of approximately 29% (8).

Treatment for GTM typically involves a multimodal approach, combining surgery, radiotherapy, and chemotherapy (9). However, due to the rarity and aggressive nature of GTM, treatment options are limited, and outcomes remain poor (10). GTM differs significantly from cutaneous melanoma (CM) in clinical presentation, pathological features, treatment response, and molecular characteristics, highlighting the need for tailored therapeutic approaches (11).

In recent years, the advent of targeted therapies and immunotherapies has begun to reshape the treatment landscape for GTM. Traditionally, surgery has been the cornerstone of treatment (6). However, advances in molecular research on cutaneous melanoma has led to the identification of key mutations, such as those in the *BRAF, NRAS*, and *NF1* genes (12). Similarly, mutations in *BRAF* and *KIT* have also been discovered in vulvar and vaginal melanomas, opening the door for the application of targeted therapies (6). At the same time, immunotherapy has also made significant progress, particularly with inhibitors targeting programmed cell death protein 1 (PD-1)/Programmed death-ligand 1 (PD-L1) and Cytotoxic T-lymphocyte-associated antigen 4 (CTLA-4) inhibitors, which have shown promising results in the treatment of cutaneous melanomas (13).

Despite these advances, the use of immunotherapy for GTM remains in its early stages and is primarily documented in case reports. Given the rarity and aggressive nature of this disease, there is a critical need for further research and well-designed clinical trials to assess the efficacy and safety of immune checkpoint inhibitors (ICIs) in this patient population. To contribute to the expanding body of knowledge, we conducted a retrospective analysis of patients with newly diagnosed or recurrent GTM treated at West China Second University Hospital from January 2019 to September 2024. By evaluating treatment outcomes and patient responses to ICIs, this study aims to provide preliminary evidence that may inform future large-scale clinical trials and guide the development of more effective therapeutic strategies for GTM.

## 2 Materials and methods

### 2.1 Study population

This retrospective study included all female patients diagnosed with GTM between January 2019 and September 2024 at West China Second University Hospital. The inclusion criteria were as follows: (1) histologically confirmed diagnosis of female genital malignant melanoma, and (2) receipt of surgery or any other therapeutic intervention. Patients were excluded if they met any of the following criteria: (1) age under 18 years, (2) a history of autoimmune disease, or (3) did not receive treatment.

Data on patient demographics, clinical examinations, tumor characteristics, surgical details, pathology results, and systemic treatments were collected from the hospital’s electronic medical record system. Tumor staging was standardized for all patients according to the 8th edition of the American Joint Committee on Cancer (AJCC) staging system to ensure consistency in the analysis. Follow-up information was obtained either from the outpatient medical record system or through telephone interviews. Progression-free survival (PFS) was defined as the time from diagnosis to the first clinically confirmed recurrence or death from the disease, while overall survival (OS) was calculated from the date of diagnosis to either the date of death or the last follow-up.

### 2.2 Statistical analysis

Kaplan-Meier survival curves were used to estimate hazard ratios for PFS and OS. Multivariate analysis was performed using Cox proportional-hazards regression models to identify significant predictors of PFS and OS. All statistical tests were two-sided, with a p-value of <0.05 considered statistically significant. Data management was conducted using Microsoft Excel, and statistical analyses were performed using SPSS software, version 25.0 (IBM Corp., Armonk, NY, USA).

### 2.3 Ethics approval

The study was approved by the Ethics Committee of West China Second University Hospital. All procedures adhered to the ethical guidelines and regulations of the committee. Due to the retrospective nature of the study, informed consent was waived.

## 3 Results

### 3.1 Patient characteristics

This study included a total of 45 patients (**Table 1**), with a median age of 55 years. Seven patients had a documented family history of malignancies among first-degree relatives, including liver cancer (2 cases), lung cancer (1 case), esophageal cancer (1 case), gastric cancer (1 case), rectal cancer (1 case), and small-cell neuroendocrine carcinoma (1 case). Cervical liquid-based cytology testing (LCT) was performed in 20 patients, of whom 9 exhibited pathological abnormalities. Cervical human papillomavirus (HPV) testing was conducted in 18 patients, revealing no infection in 13 cases. Among the 5 HPV-positive patients (3 vulvar melanoma and 2 vaginal melanoma), 2 tested positive for HPV56, and 1 each for HPV16, HPV52, and HPV58. Among the 45 patients, 24 (53.3%) were diagnosed with vaginal melanoma, 18 (40.0%) with vulvar melanoma, and 3 (6.7%) with cervical melanoma. At the time of initial diagnosis, 3 patients (6.5%) were classified as AJCC stage I, 32 (71.1%) as stage II, 9 (20.0%) as stage III, and 1 (2.2%) as stage IV.

**Table 1:** Clinicopathological characteristics of gynecologic tract melanoma.

Clinicopathological characteristics	GTMs(n)	n%
**Age, median(range),y**	55(31-79)	
Age		
≤55	23	51.1
>55	22	48.9
LDH		
≤230	38	84.4
>230	6	13.3
NA	1	2.3
FHC		
Yes	7	15.6
No	38	84.4
LCT		
Positive	9	20.0
Negative	11	24.4
NA	25	55.6
HPV		
Positive	5	11.1
Negative	13	28.9
NA	27	60.0
Subtype		
PMVa	24	53.3
PMVu	18	40.0
PMC	3	6.7
AJCC stage*		
IA	1	2.2
IB	2	4.4
IIA	5	11.2
IIB	26	57.8
IIC	1	2.2
III	9	20.0
IV	1	2.2
Tumor size, mm		
≤1	3	6.7
>1, ≤2	0	0.0
>2, ≤4	6	13.3
>4	36	80.0
Ulceration		
Yes	2	4.4
No	43	95.6
Ki-67		
≤75%	37	82.2
>75%	4	8.9
NA	4	8.9
RLN metastasis		
Yes	9	20.0
No	18	40.0
NA	18	40.0
Cuff involvement		
Involved	12	26.7
Uninvolved	28	62.2
NA	5	11.1
Distant metastasis		
Yes	1	2.2
No	44	97.8
Surgical approaches		
TWLE	15	33.3
TWLE + CLND	13	28.9
RH + BSO	2	4.4
RH + CLND + BSO	14	31.1
Non-surgical treatment	1	2.3
Neoadjuvant treatment		
Yes	5	11.1
No	40	88.9
Recurrences		
Yes	17	37.8
No	25	55.6
NA	3	6.6
Recurrence site		
Distant	3	6.7
Regional	5	11.1
NA	9	20.0
Outcome		
Alive	27	60.0
Died	14	31.1
NA	4	8.9

GTM, gynecologic tract melanoma; LDH, lactate dehydrogenase; FHC, family history of cancer; LCT, liquid-based cytologic test; HPV, human papillomavirus; PMVa, primary melanoma of vagina; PMVu, primary melanomas of vulva; PMC, primary melanomas of cervix; RLN, regional lymph nodes; NA, not available; TWLE, tumor wide local excision; CLND, complete lymph node dissection; RH, Radical Hysterectomy; BSO, bilateral salpingo-oophorectomy.

* We use American Joint Committee on Cancer (AJCC) version 8 staging system.

### 3.2 Treatment approaches for new diagnosed GTM

Four patients received neoadjuvant chemotherapy prior to surgery. Of the total cohort, 44 patients (97.8%) underwent surgery as their initial treatment. Tumor wide local excision (TWLE) served as the standard procedure for vulvar melanoma, with complete lymph node dissection (CLND) performed selectively based on tumor size and imaging findings indicating lymph node enlargement. Similar to vulvar malignant melanoma, vaginal malignant melanoma in the lower one-third of the vagina was treated with TWLE or TWLE combined with CLND. In contrast, vaginal malignant melanoma in the upper one-third of the vagina was managed using the same surgical approach as cervical malignant melanoma, namely radical hysterectomy (RH) and bilateral salpingo-oophorectomy (BSO), with or without CLND. Specifically, 15 patients (33.3%) underwent TWLE alone, 13 (28.9%) received TWLE combined with CLND, 2 (4.4%) had RH and BSO due to poor general condition, and 14 (31.1%) underwent RH in combination with both CLND and BSO.

Postoperatively, 34 patients (75.6%) received adjuvant therapy (**Table 2**). Among them, 11 patients (24.4%) underwent chemotherapy, with regimens including dacarbazine combined with cisplatin, temozolomide with cisplatin, and dacarbazine monotherapy. Five patients (11.1%) received adjuvant immunotherapy, with single-agent pembrolizumab or toripalimab. Additionally, 2 patients (4.4%) received adjuvant radiotherapy, while 1 patient (2.2%) was treated with targeted therapy using axitinib. Combination therapies were also administered, including chemotherapy with ICIs in 9 patients (20.0%), chemotherapy with radiotherapy in 3 patients (6.7%), ICIs combined with chemotherapy and targeted therapy in 1 patient (2.2%), and ICIs combined with chemotherapy and radiotherapy in 2 patients (4.4%).

**Table 2:** Adjuvant therapy details among patients presenting with primary disease.

Characteristics(N=45)	GTMs(n)	n%
No adjuvant therapy	11	24.4
Adjuvant chemotherapy	11	24.4
Dacarbazine and Cisplatin	3	6.7
Temozolomide and Cisplatin	5	11.1
Dacarbazine	1	2.2
NA	2	4.4
Adjuvant immunotherapy	5	11.1
Pembrolizumab	3	6.7
Toripalimab	1	2.2
NA	1	2.2
Adjuvant radiotherapy	2	4.4
Adjuvant targeted therapy	1	2.2
Axitinib	1	2.2
Chemotherapy and immunotherapy	9	20.0
Pembrolizumab, dacarbazine and cisplatin	5	11.1
Pembrolizumab and cisplatin	1	2.2
Pembrolizumab, cisplatin and paclitaxel	1	2.2
NA	2	4.4
Chemotherapy and radiotherapy	3	6.7
Temozolomide, cisplatin and radiotherapy	1	2.2
Etoposide, ifosfamide and radiotherapy	1	2.2
NA	1	2.2
Immunotherapy, chemotherapy and targeted therapy	1	2.2
Pembrolizumab, dacarbazine, cisplatin and bevacizumab	1	2.2
Immunotherapy, chemotherapy and radiotherapy	2	4.4
Toripalimab, carboplatin, paclitaxel and radiotherapy	1	2.2
Unknown type of immunotherapy, dacarbazine, cisplatin and radiotherapy	1	2.2

NA, not available.

### 3.3 Clinical outcomes

In a cohort of 45 patients, 14 patients died within five years of diagnosis, with a median survival time of 24.00 months (IQR: 5.50–36.75). Among the 27 surviving patients, the median follow-up time was 23.00 months (IQR: 13.00–47.00). The 1-, 3-, and 5-year survival rates were 87%, 63%, and 31%, respectively. Regarding disease progression, 21 out of 45 patients experienced progression within five years, with a median PFS of 9.00 months (IQR: 3.50–15.50). The remaining 21 patients had a median follow-up time of 22.00 months (IQR: 12.50–47.50). The 1-, 3-, and 5-year progression rates were 32%, 49%, and 72%, respectively.

### 3.4 Prognostic factors

**Figure 1:**
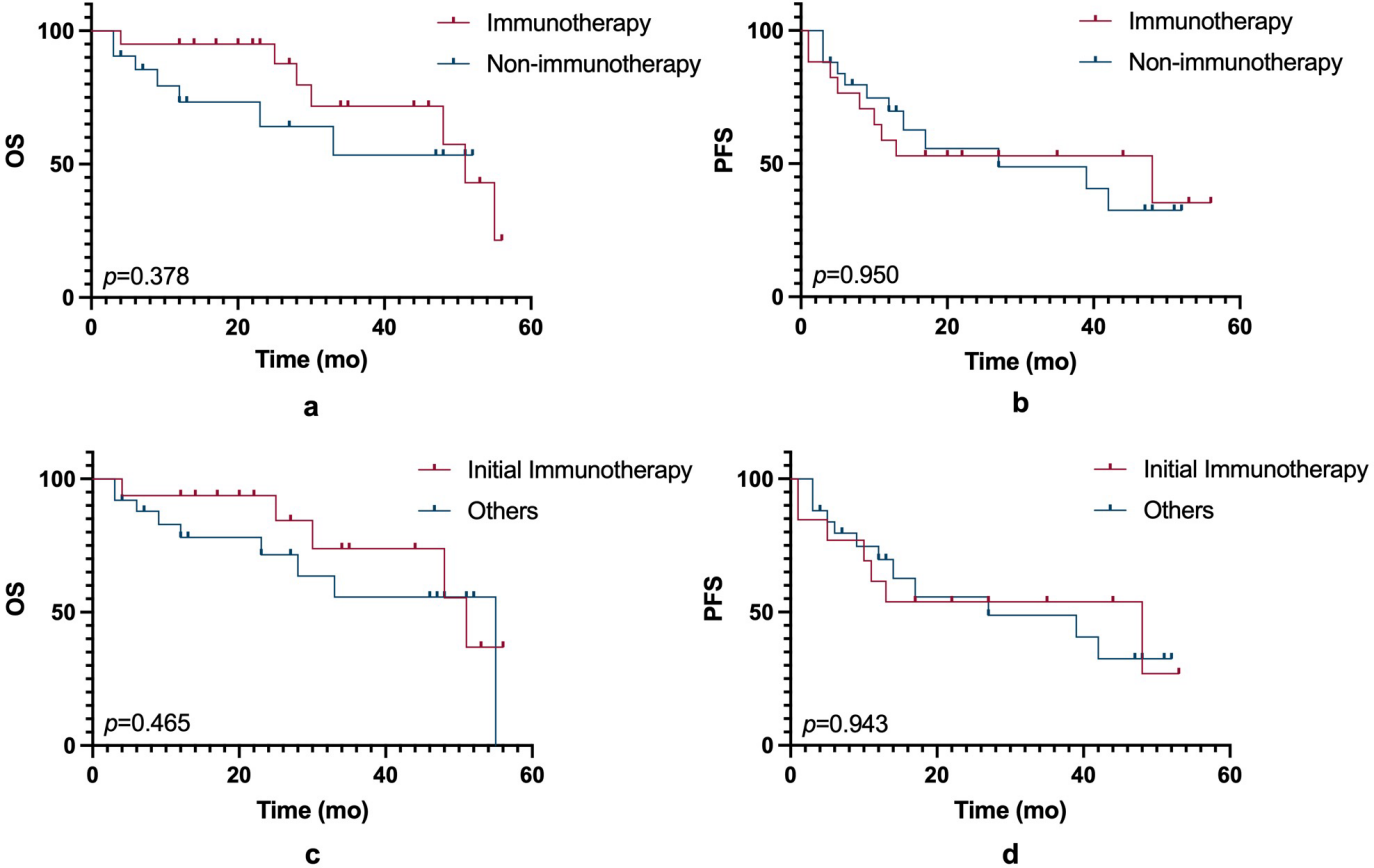
OS and PFS comparisons between immunotherapy and non-immunotherapy groups. **(a)** Kaplan-Meier curves showing OS between patients treated with immunotherapy and those without immunotherapy (p = 0.378). **(b)** Kaplan-Meier curves of PFS between the immunotherapy and non-immunotherapy groups (p = 0.950). **(c)** Comparison of OS between patients receiving initial immunotherapy and those treated with other therapies (p = 0.465). **(d)** PFS between patients receiving initial immunotherapy and other treatment groups (p = 0.943). Statistical significance is indicated by the p-values in each panel.

**Figure 2:**
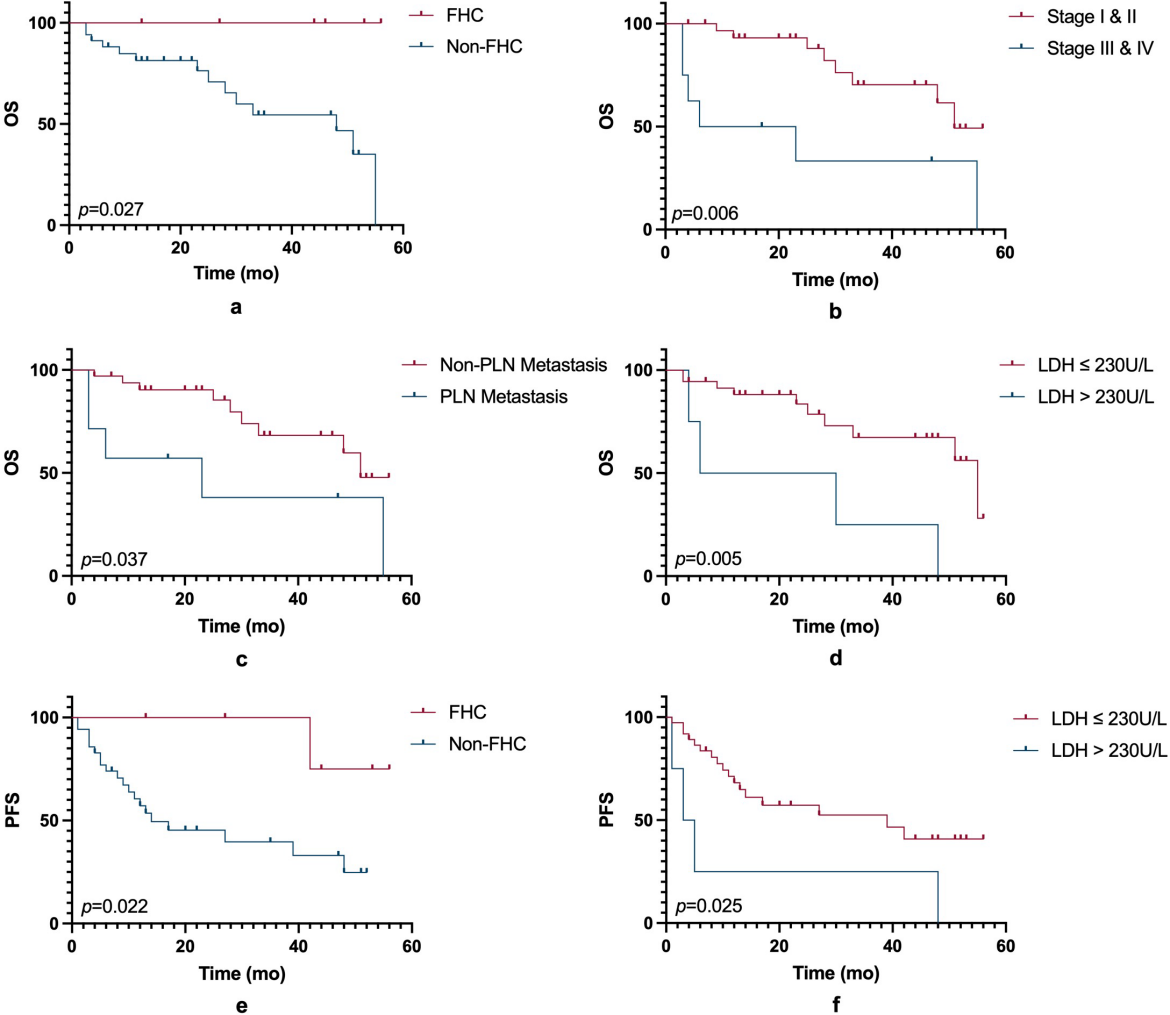
OS and PFS based on clinical and biological factors. **(a)** Kaplan-Meier curves for OS comparing patients with a family history and those without (p = 0.027). **(b)** OS curves comparing early-stage (Stage I & II) and advanced-stage (Stage III & IV) patients (p = 0.006). **(c)** OS curves comparing patients with and without regional lymph node (RLN) metastasis (p = 0.037). **(d)** OS curves comparing patients with serum LDH levels ≤230 U/L and >230 U/L (p = 0.005). **(e)** PFS curves comparing patients with a family history and those without (p = 0.022). **(f)** PFS curves comparing patients with LDH levels ≤230 U/L and >230 U/L (p = 0.025). Statistical significance is indicated by the p-values in each panel. FHC, family history of cancer; PLN, pelvic lymph node; LDH, lactate dehydrogenase.

In this small-sample study, ICIs did not significantly improve PFS or OS compared to non-ICIs treatment. (**Figure 1**). However, other prognostic factors were identified (**Figure 2**). **Table 3** summarizes clinicopathological characteristics associated with PFS and OS in GTMs. Patients with a family history of cancer (FHC) had longer PFS than those without (52.5 months vs. 25.3 months, p = 0.022). Similarly, initial lactate dehydrogenase (LDH) level ≤230 U/L were associated with improved PFS compared to >230 U/L (32.6 months vs. 14.3 months, p = 0.025). Though not statistically significant, trends indicated better PFS in patients with AJCC stage I-II compared to stage III-IV (34.0 months vs. 19.5 months, p = 0.079) and in those with tumor diameter ≤4 mm compared to >4 mm (27.4 months vs. 48.2 months, p = 0.084). However, multivariate analysis did not identify FHC (p = 0.064) or LDH level (p = 0.122) as independent prognostic factors for PFS.

**Table 3:** Univariate and multivariate survival analysis of associated clinicopathological characteristics with PFS and OS in GTMs.

Variables	Categories	PFS			OS		
		Univariate P Value	Multivariate P Value	Multivariate HR (95%CI)	Univariate P Value	Multivariate P Value	Multivariate HR (95%CI)
Age(y)	≤55	0.686			0.697		
	>55						
FHC	Yes	0.022	0.064	0.019-1.120	0.027†		
	No						
AJCC stage*	I & II	0.079			0.006	0.102	0.009-1.540
	III & IV						
Tumor site	Vulva	0.294			0.083		
	Vagina						
PLN metastasis	Yes	0.241			0.037	0.548	0.031-6.361
	No						
Ki-67	≤75%	0.582			0.075		
	>75%						
LDH	≤230	0.025	0.122	0.134-1.269	0.005	0.034	0.059-0.894
	>230						
Cuff involvement	Yes	0.459			0.698		
	No						
Tumor size(mm)	≤4	0.084			0.236		
	>4						
Surgical approaches	CLND	0.457			0.973		
	non-CLND						
Adjuvant treatment	Yes	0.524			0.099		
	No						
Immunotherapy	Yes	0.943			0.378		
	No						
Initial immunotherapy	Yes	0.950			0.465		
	No						

PFS, progression-free survival; OS, overall survival; FHC, family history of cancer; PLN, pelvic lymph node; LDH, lactate dehydrogenase; CLND, complete lymph node dissection.

* We use American Joint Committee on Cancer (AJCC) version 8 staging system.

† FHC was collinearity with other factors and therefore was not included in Cox regression model.

For OS, patients with FHC had better OS compared to those without (p = 0.027). OS was also prolonged in AJCC stage I-II compared to stage III-IV (45.4 months vs. 24.2 months, p = 0.006) and in those without pelvic lymph node (PLN) metastasis (44.2 months vs. 27.0 months, p = 0.037).An initial serum LDH level ≤230 U/L was strongly associated with improved OS (43.4 months 22.0 months, p = 0.005). Although not statistically significant, trends suggested longer OS in patients with vulvar melanoma compared to vaginal melanoma (47.7 months vs. 35.1 months, p = 0.083), and those receiving adjuvant therapy compared to those who did not (44.6 months vs. 29.3 months, p = 0.099). Subgroup analysis further indicated improved OS in AJCC stage I-II vulvar melanoma patients (p = 0.017). Due to collinearity, FHC was excluded from the multivariate analysis of OS. The analysis revealed that AJCC stage (p = 0.102) and PLN metastasis (p = 0.548) were not independent prognostic factors for OS, whereas LDH level (p = 0.034) was identified as an independent prognostic factor.

### 3.5 Therapy for recurrent GTMs

Seventeen patients experienced recurrence during follow-up, including 6 with vulvar melanoma, 10 with vaginal melanoma, and 1 with cervical melanoma (**Table 4**). Among them, 5 patients had local recurrence, 3 had distant recurrence, and recurrence details were unavailable for 9 patients.

**Table 4:** Therapy for recurrent GTMs.

No	Age	Tumor site	Recurrence site	Surgery	Chemotherapy	Immunotherapy	Radiotherapy	Outcome	PFS(m)	OS (m)
1	67	Vulva	NA	NA				NA	8	15
3	51	Vulva	Regional	Yes	Yes	No	No	Alive	4	34
5	70	Vulva	Distant	No	Yes	Yes	No	Died	39	55
7	48	Vagina	Regional	Yes	No	Yes	No	Died	13	51
8	43	Vagina	NA	NA				Died	1	4
10	58	Vagina	Regional	Yes	Yes	No	No	Died	5	30
11	59	Vagina	NA	NA				Died	1	25
14	51	Vagina	NA	No	Yes	Yes	Yes	Alive	42	46
15	53	Vagina	NA	No	Yes	Yes	No	Died	14	28
16	31	Vulva	Regional	Yes	Yes	Yes	No	Alive	10	14
21	51	Vulva	NA	NA				Died	17	23
25	73	Vagina	Distant	No	No	No	No	Alive	27	48
28	46	Vagina	NA	NA				Died	6	9
31	75	Vagina	NA	No	No	No	Yes	Died	9	33
32	74	Vulva	NA	NA				Died	3	6
36	36	Cervix	Regional	No	No	Yes	No	Alive	5	23
42	49	Vagina	Distant	Yes	No	No	No	Alive	11	12

PFS, progression-free survival; OS, overall survival; NA, not available.

Regarding treatment for recurrence, 1 patient received surgery, another received ICIs, and another received radiotherapy as part of their relapse treatment; 2 patients underwent surgery and chemotherapy; 1 patient received surgery and ICIs; and 2 patients received chemotherapy andICIs. Additionally, 1 patient received surgery, chemotherapy, andICIs, while another underwent multiple courses of radiotherapy, chemotherapy, and ICIs. One patient did not receive any treatment, and the specific treatment details for 6 patients were unknown.

During the follow-up period, 1 patient was lost to follow-up, and 10 patients died. The median PFS for patients with recurrence was 9 months (IQR: 4.5–15.5), and the median OS was 25 months (IQR: 13.0–40.0).

### 3.6 ICIs for GTM patients

A total of 21 patients received ICIs (**Table 5**), including 10 with vulvar melanoma, 7 with vaginal melanoma, and 4 with cervical melanoma. Seventeen patients received postoperative adjuvant therapy, while 6 had recurrent disease, including 2 who underwent ICIs during both initial and recurrent treatments. Among the ICIs regimens, 11 patients received pembrolizumab, 6 received toripalimab, 1 received camrelizumab, and the specific immunotherapy agents were unknown in 3 cases.

**Table 5:** Immunotherapy for 21 GTM patients.

No	Age	Tumor site	Primary/Recurrence	AJCC stage*	Recurrence type	Immunotherapy	Other treatment	ORR	Outcome	PFS(m)	OS(m)
1	67	Vulva	Primary	IIB		Pembrolizumab	TWLE + CLND + Chemotherapy	PD	NA	8	15
2	47	Vulva	Primary	IIA		Pembrolizumab	TWLE + Chemotherapy	CR	Alive	56	56
3	51	Vulva	Primary	IIB		Pembrolizumab	TWLE + CLND + Chemotherapy	PD	Alive	4	34
4	57	Vulva	Primary	IIB		Pembrolizumab	TWLE	CR	Alive	20	20
5	70	Vulva	Recurrence		Distant	Toripalimab	Chemotherapy	PD	Died	39	55
6	79	Cervix	Primary	IIB		Pembrolizumab	Neoadjuvant therapy + TWLE + BSO	CR	Died	48	48
7	48	Vagina	Primary	IIC		Pembrolizumab	TWLE + Chemotherapy	PD	Died	13	51
			Recurrence		Regional	Pembrolizumab	TWLE	PR			
8	43	Vagina	Primary	IV		Pembrolizumab	Chemotherapy	PD	Died	1	4
9	55	Vagina	Primary	IIB		Pembrolizumab	TWLE + CLND + BSO + Chemotherapy	CR	Alive	35	35
10	58	Vagina	Primary	IIB		Pembrolizumab	TWLE + CLND + BSO + Chemotherapy	PD	Died	5	30
11	59	Vagina	Primary	IIB		Pembrolizumab	TWLE + Chemotherapy + Targeted therapy	PD	Died	25	25
12	50	Vagina	Primary	IIB		Toripalimab	Neoadjuvant therapy + TWLE + Chemotherapy + Radiotherapy	CR	Alive	27	27
14	51	Vagina	Recurrence		NA	Toripalimab	Chemotherapy + Radiotherapy	NA	Alive	42	46
15	53	Vagina	Recurrence		NA	Toripalimab	Chemotherapy	PD	Died	14	28
16	31	Vulva	Primary	IIB		Toripalimab	TWLE + CLND + Chemotherapy	PR	Alive	10	14
			Recurrence		Distant	Toripalimab	Surgery + Chemotherapy	PD			
19	43	Vulva	Primary	IIB		NA	TWLE + Chemotherapy + Radiotherapy	CR	Alive	53	53
27	65	Vulva	Primary	IIB		NA	TWLE + CLND	CR	Alive	44	44
35	67	Vulva	Primary	IIB		Pembrolizumab	TWLE + CLND	CR	Alive	22	22
36	36	Cervix	Recurrence		Regional	Cardunolizumab	Disitamab Vedotin	NA	Alive	5	23
37	44	Vulva	Primary	III		Toripalimab	TWLE + CLND	CR	Alive	17	17
42	49	Vagina	Primary	IIB		NA	TWLE + CLND + BSO + Chemotherapy	PD	Alive	11	12

ORR, objective response rate; PFS, progression-free survival; OS, overall survival; TWLE, tumor wide local excision; CLND, complete lymph node dissection; BSO, bilateral salpingo-oophorectomy; CR, complete response; PR, partial response; PD, progressive disease; NA, not available.

*We use American Joint Committee on Cancer (AJCC) version 8 staging system.

Among these 21 patients, excluding two recurrent cases for whom tumor size changes could not be tracked during follow-up, the remaining 19 had evaluable responses. Of these, 9 achieved complete response (CR) and 2 achieved partial response (PR), resulting in an overall objective response rate (ORR) of 52.4%. Additionally, 1 patient lacked survival data, and 7 patients had died, leading to a mortality rate of 35% among GTM patients who received ICIs. When stratified by treatment setting, among the 17 patients who received postoperative adjuvant immunotherapy, 9 achieved CR and 1 achieved PR, yielding an ORR of 58.8%. In contrast, among the 6 patients treated for recurrent disease, 1 achieved PR, 3 experienced progressive disease (PD), and 2 were lost to follow-up, resulting in an ORR of 25%.

## 4 Discussion

### 4.1 Limitations of ICIs in GTM

This study did not observe a significant survival benefit of ICIs in patients with GTM compared to non-ICIs treatment. Although ICIs have demonstrated efficacy in cutaneous melanoma (CM) and some MM (14–16), their role in GTM remains limited. Among the 21 patients who received ICIs in our cohort, 17 were treated in the initial setting, and 6 received ICIs after recurrence. However, the median OS among ICI-treated patients was only 25 months, with more than half eventually succumbing to the disease. This finding aligns with an analysis of the Surveillance, Epidemiology, and End Results (SEER) database, which included 1,863 melanoma patients and found no clear survival benefit of ICIs in GTM (15). While Boer et al. reported improved survival with ICIs in unresectable melanomas (17), other studies have yielded inconsistent results (18). Furthermore, despite evidence suggesting that radiotherapy combined with immunotherapy may exert a synergistic anti-tumor effect (17), only two patients in our study received this combination, making it difficult to evaluate its efficacy.

The limited efficacy of ICIs in GTM may be attributed to its distinct biological characteristics. Previous studies, including EORTC 18071, CheckMate-238, and KEYNOTE-054, have demonstrated the benefit of adjuvant ICIs in improving recurrence-free survival (RFS) in stage III CM patients (19). Notably, anti-CTLA-4 drugs have not been shown to improve ORR in GTM, unlike their efficacy in melanomas of the naso-oral mucosa (20). This discrepancy may stem from the unique immune microenvironment of GTM, which differs significantly from that of CM (21). Moreover, due to its rarity and the lack of prospective randomized controlled trials (RCTs), current knowledge about ICIs in GTM is primarily derived from case reports and small retrospective studies, highlighting the need for larger, multicenter studies (13, 22).

### 4.2 Exploring novel immunotherapy approaches

Recent advances in immunotherapy have introduced promising strategies for melanoma treatment. For example, mRNA vaccines combined with PD-1 inhibitors may enhance RFS in high-risk melanoma patients. The KEYNOTE-942 study demonstrated that mRNA-4157 (V940) plus pembrolizumab significantly prolonged RFS compared to pembrolizumab monotherapy (23). Additionally, tumor-infiltrating lymphocyte (TIL) therapy has shown efficacy in certain melanoma subtypes, with studies indicating a higher degree of TIL infiltration is associated with better prognosis (24, 25). Furthermore, as presented by Grigoleit et al. at the 2023 ESMO (European Society for Medical Oncology) Congress in Madrid, results from the C-144-01 study involving 15 patients with mucosal melanoma treated with lifileucel were encouraging, demonstrating an ORR of 50%, with the median duration of response not yet reached at the time of analysis. Although these therapies have shown potential in CM, their role in GTM remains largely unexplored, warranting further investigation.

### 4.3 The role of neoadjuvant therapy

In our study, only four patients received neoadjuvant therapy, limiting the ability to assess its effectiveness. Similarly, a meta-analysis of eight RCTs found its efficacy in stage III and IV melanoma remains uncertain, with low-quality evidence for OS and DFS improvement (26). Historically, data did not confirm the superiority of neoadjuvant therapy over surgery followed by adjuvant therapy.

However, recent landmark trials have begun to shift this perspective. The SWOG S1801 trial (NEJM, 2023) demonstrated that in resectable stage III–IV melanoma, neoadjuvant pembrolizumab followed by surgery and adjuvant therapy significantly improved event-free survival (EFS) compared to adjuvant therapy alone (2-year EFS: 72% vs. 49%) (27). More strikingly, the phase 3 NADINA trial (NEJM, 2024) showed that two cycles of neoadjuvant nivolumab plus ipilimumab, followed by surgery and response-adapted adjuvant therapy, led to a 12-month EFS of 83.7% compared to 57.2% in the adjuvant-only group, with a major pathological response observed in 59% of patients (28). These findings highlight the immunological advantages of administering ICIs in the neoadjuvant setting, where the presence of the intact tumor may help prime a more effective systemic anti-tumor immune response. For advanced GTM, these results underscore the urgent need for prospective studies to explore the feasibility, safety, and potential efficacy of neoadjuvant immunotherapy or rational combination regimens. Such approaches may hold promise for improving outcomes in this challenging disease.

### 4.4 Challenges in treating recurrent GTM

Recurrent GTM remains a major therapeutic challenge, with limited treatment options. In our cohort, 17 patients experienced recurrence, and among the 6 who received ICIs with or without chemotherapy or radiotherapy, ORR was only 25%. This suggests that ICIs alone or in combination with chemotherapy or radiotherapy have limited efficacy. Novel immunotherapeutic approaches such as mRNA vaccines and TIL therapy, or the combination of ICIs with targeted therapy, may offer more promising strategies to improve outcomes in recurrent GTM.

In our study, two patients underwent immune rechallenge after disease recurrence, with one surviving 38 months and the other still under follow-up. Research suggests that switching to a different ICI may restore anti-tumor response, whereas continuing the same ICI post-progression may increase immune-related adverse events and reduce ORR (29–31). While current data are primarily derived from non-small cell lung cancer and renal cell carcinoma (29), further studies are needed to evaluate immune rechallenge strategies in GTM.

### 4.5 Prognostic factors in GTM

In this study, LDH levels were significant prognostic factors. Patients with LDH levels below 230 U/L had improved OS, suggesting that LDH could serve as a prognostic biomarker for GTM, similar to its role in metastatic melanoma (32, 33). Additionally, Patients with early-stage disease (AJCC I-II) had significantly better OS than those with advanced-stage disease, particularly in vulvar melanoma, which is consistent with previous studies (19). Lymph node metastasis also strongly influenced survival outcomes. A study of 1,863 GTM patients demonstrated that lymph node status is an independent predictor of survival (19, 34). Although we did not observe a significant impact of AJCC stage and PLN status on PFS, likely due to the limited sample size, comprehensive lymph node assessment remains crucial for staging and treatment planning.

Interestingly, FHC was associated with longer PFS, suggesting potential genetic or immunological influences. Previous studies have shown that patients with FHC may derive greater benefit from PD-1/PD-L1 therapy, possibly due to genetic mutations enhancing immune recognition (35). Further large-scale studies are needed to validate this hypothesis.

## 5 Limitation

This study has several limitations. As a single-center retrospective study with a small sample size, the findings may be influenced by selection bias and may not be fully generalizable. The heterogeneous treatment regimens and short follow-up period further limit the ability to assess the true impact of ICIs and long-term survival outcomes. Additionally, the lack of molecular and immune profiling data restricts insight into potential biomarkers that could predict treatment response. Finally, the absence of randomized controlled trials (RCTs) prevents definitive conclusions about the efficacy of ICIs in GTM. Despite these limitations, this study provides valuable preliminary data, emphasizing the need for larger, multicenter studies to optimize treatment strategies.

## 6 Conclusion

Our study indicates that despite the widespread use of ICIs in GTM patients, their survival benefit remains unclear. The rarity of GTM and the lack of prospective studies limit our understanding of optimal treatment strategies. Future research should focus on large-scale, multicenter trials to refine therapeutic approaches. Additionally, novel treatments such as mRNA vaccines, TIL therapy, and neoadjuvant immunotherapy have shown promise in CM and warrant further evaluation in GTM. LDH levels, AJCC stage, and lymph node status remain key prognostic factors, emphasizing the importance of individualized treatment strategies for this aggressive malignancy.

## Data Availability

The raw data supporting the conclusions of this article will be made available by the authors, without undue reservation.

## References

[1] Pang Y Yuan H Ren A Zhang S Liu P. Primary Malignant melanoma of the female genital tract synchronously involving the vulva and uterine cervix: a case report. Med (Baltimore). (2019) 98:e16366. doi: 10.1097/MD.0000000000016366 PMC6708980 31348237

[2] Chang AE Karnell LH Menck HR. The National Cancer Data Base report on cutaneous and noncutaneous melanoma: a summary of 84,836 cases from the past decade. Am Coll Surgeons Commission Cancer Am Cancer Society. Cancer. (1998) 83:1664–78. doi: 10.1002/(SICI)1097-0142(19981015)83:8<1664::AID-CNCR23>3.0.CO;2-G 9781962

[3] Vaysse C Pautier P Filleron T Maisongrosse V Rodier J-F Lavoue V. A large retrospective multicenter study of vaginal melanomas: implications for new management. Melanoma Res. (2013) 23:138–46. doi: 10.1097/CMR.0b013e32835e590e 23449321

[4] Mert I Semaan A Winer I Morris RT Ali-Fehmi R. Vulvar/vaginal melanoma: an updated surveillance epidemiology and end results database review, comparison with cutaneous melanoma and significance of racial disparities. Int J Gynecol Cancer Off J Int Gynecol Cancer Soc. (2013) 23:1118–25. doi: 10.1097/IGC.0b013e3182980ffb 23765206

[5] Nassar KW Tan AC. The mutational landscape of mucosal melanoma. Semin Cancer Biol. (2020) 61:139–48. doi: 10.1016/j.semcancer.2019.09.013 PMC7078020 31655118

[6] Boer FL Ten Eikelder MLG Kapiteijn EH Creutzberg CL Galaal K Van Poelgeest MIE. Vulvar Malignant melanoma: pathogenesis, clinical behaviour and management: review of the literature. Cancer Treat Rev. (2019) 73:91–103. doi: 10.1016/j.ctrv.2018.12.005 30685613

[7] Guzik P Łukasiewicz M Harpula M Zając P Żmuda M Śniadecki M. Survival and treatment modalities in primary vaginal melanoma-case report and a narrative review. J Clin Med. (2024) 13:3771. doi: 10.3390/jcm13133771 38999339 PMC11242499

[8] Min A Fu A Huang M Wang H Chen H. Primary Malignant melanoma of the cervix: an integrated analysis of case reports and series. Front Oncol. (2022) 12:913964. doi: 10.3389/fonc.2022.913964 35814437 PMC9258497

[9] Dobrică E-C Vâjâitu C Condrat CE Crețoiu D Popa I Gaspar BS. Vulvar and vaginal melanomas—the darker shades of gynecological cancers. Biomedicines. (2021) 9:758. doi: 10.3390/biomedicines9070758 34209084 PMC8301463

[10] Mitra D Farr M Nagarajan P Ho J Bishop AJ Jhingran A. Gynecologic tract melanoma in the contemporary therapeutic era: High rates of local and distant disease progression. Gynecol Oncol. (2022) 167:483–9. doi: 10.1016/j.ygyno.2022.09.026 PMC11622147 36229264

[11] Hou JY Baptiste C Hombalegowda RB Tergas AI Feldman R Jones NL. Vulvar and vaginal melanoma: a unique subclass of mucosal melanoma based on a comprehensive molecular analysis of 51 cases compared with 2253 cases of nongynecologic melanoma. Cancer. (2017) 123:1333–44. doi: 10.1002/cncr.30473 28026870

[12] Moran JMT. Identification of fusions with potential clinical significance in melanoma. Mod Pathol. (2022) 35:1837–47. doi: 10.1038/s41379-022-01138-z 35871080

[13] Indini A Di Guardo L Cimminiello C Lorusso D Raspagliesi F Del Vecchio M. Investigating the role of immunotherapy in advanced/recurrent female genital tract melanoma: a preliminary experience. J Gynecol Oncol. (2019) 30:e94. doi: 10.3802/jgo.2019.30.e94 31576688 PMC6779609

[14] Ostby SA Daniel S Kalogera E De Vitis L Fought AJ McGree ME. Treatment outcomes of vulvar and vaginal melanoma at an NCCN institution between 1993 and 2021. Gynecol Oncol Rep. (2024) 55:101483. doi: 10.1016/j.gore.2024.101483 39247489 PMC11379566

[15] Wohlmuth C Wohlmuth-Wieser I. Vulvar melanoma: molecular characteristics, diagnosis, surgical management, and medical treatment. Am J Clin Dermatol. (2021) 22:639–51. doi: 10.1007/s40257-021-00614-7 PMC8421300 34125416

[16] Ladwa A Elghawy O Kaur V Hernandez E. Single institution experience with immune checkpoint inhibitors in vulvar and vaginal melanomas. Obstet Gynecol Int. (2024) 2024:7327692. doi: 10.1155/2024/7327692 39166179 PMC11335409

[17] Boer FL Ten Eikelder MLG Van Geloven N Kapiteijn EH Gaarenstroom KN Hughes G. Evaluation of treatment, prognostic factors, and survival in 198 vulvar melanoma patients: implications for clinical practice. Gynecol Oncol. (2021) 161:202–10. doi: 10.1016/j.ygyno.2021.01.018 33514483

[18] Albert A Lee A Allbright R Vijayakumar S. Vulvar melanoma: an analysis of prognostic factors and treatment patterns. J Gynecol Oncol. (2020) 31:e66. doi: 10.3802/jgo.2020.31.e66 32808496 PMC7440982

[19] Wohlmuth C Wohlmuth-Wieser I May T Vicus D Gien LT Laframboise S. Malignant melanoma of the vulva and vagina: A US population-based study of 1863 patients. Am J Clin Dermatol. (2020) 21:285–95. doi: 10.1007/s40257-019-00487-x PMC7125071 31784896

[20] Dimitriou F Namikawa K Reijers ILM Buchbinder EI Soon JA Zaremba A. Single-agent anti-PD-1 or combined with ipilimumab in patients with mucosal melanoma: an international, retrospective, cohort study. Ann Oncol. (2022) 33:968–80. doi: 10.1016/j.annonc.2022.06.004 35716907

[21] Falcone I Conciatori F Bazzichetto C Ferretti G Cognetti F Ciuffreda L. Tumor microenvironment: implications in melanoma resistance to targeted therapy and immunotherapy. Cancers. (2020) 12:2870. doi: 10.3390/cancers12102870 33036192 PMC7601592

[22] Postow MA Luke JJ Bluth MJ Ramaiya N Panageas KS Lawrence DP. Ipilimumab for patients with advanced mucosal melanoma. Oncologist. (2013) 18:726–32. doi: 10.1634/theoncologist.2012-0464 PMC4063400 23716015

[23] Weber JS Carlino MS Khattak A Meniawy T Ansstas G Taylor MH. Individualised neoantigen therapy mRNA-4157 (V940) plus pembrolizumab versus pembrolizumab monotherapy in resected melanoma (KEYNOTE-942): a randomised, phase 2b study. Lancet. (2024) 403:632–44. doi: 10.1016/S0140-6736(23)02268-7 38246194

[24] Azimi F Scolyer RA Rumcheva P Moncrieff M Murali R McCarthy SW. Tumor-infiltrating lymphocyte grade is an independent predictor of sentinel lymph node status and survival in patients with cutaneous melanoma. J Clin Oncol. (2012) 30:2678–83. doi: 10.1200/JCO.2011.37.8539 22711850

[25] Minowa T Murata K Mizue Y Murai A Nakatsugawa M Sasaki K. Single-cell profiling of acral melanoma infiltrating lymphocytes reveals a suppressive tumor microenvironment. Sci Transl Med. (2024) 16:eadk8832. doi: 10.1126/scitranslmed.adk8832 39630887

[26] Gorry C McCullagh L O’Donnell H Barrett S Schmitz S Barry M. Cochrane Skin Group, editor. Neoadjuvant treatment for stage III and IV cutaneous melanoma. Cochrane Database Syst Rev. (2023) 17 :CD012974. doi: 10.1002/14651858.CD012974.pub2 PMC9844053 36648215

[27] Patel SP Othus M Chen Y Wright GP Yost KJ Hyngstrom JR. Neoadjuvant–adjuvant or adjuvant-only pembrolizumab in advanced melanoma. N Engl J Med. (2023) 388:813–23. doi: 10.1056/NEJMoa2211437 PMC10410527 36856617

[28] Blank CU Lucas MW Scolyer RA Van De Wiel BA Menzies AM Lopez-Yurda M. Neoadjuvant nivolumab and ipilimumab in resectable stage III melanoma. N Engl J Med. (2024) 391:1696–708. doi: 10.1056/NEJMoa2402604 38828984

[29] Inno A Roviello G Ghidini A Luciani A Catalano M Gori S. Rechallenge of immune checkpoint inhibitors: A systematic review and meta-analysis. Crit Rev Oncol Hematol. (2021) 165:103434. doi: 10.1016/j.critrevonc.2021.103434 34343657

[30] Ribas A Puzanov I Dummer R SChadendorf D Hamid O Robert C. Pembrolizumab versus investigator-choice chemotherapy for ipilimumab-refractory melanoma (KEYNOTE-002): a randomised, controlled, phase 2 trial. Lancet Oncol. (2015) 16:908–18. doi: 10.1016/S1470-2045(15)00083-2 PMC9004487 26115796

[31] Inno A Lo Russo G Salgarello M Corrao G Casolino R Galli G. The evolving landscape of criteria for evaluating tumor response in the era of cancer immunotherapy: From Karnofsky to iRECIST. Tumori. (2018) 104:88–95. doi: 10.1177/0300891618766173 29714647

[32] Bustos MA Gross R Rahimzadeh N Cole H Tran LT Tran KD. A pilot study comparing the efficacy of lactate dehydrogenase levels versus circulating cell-free microRNAs in monitoring responses to checkpoint inhibitor immunotherapy in metastatic melanoma patients. Cancers. (2020) 12:3361. doi: 10.3390/cancers12113361 33202891 PMC7696545

[33] Yamashita C Otsuka A Nomura M Honda T Kabashima K. Successful treatment of metastatic mucosal melanoma with a Del579 c-KIT mutation by imatinib after treatment of anti-PD-1 antibody. J Eur Acad Dermatol Venereol JEADV. (2019) 33:e92–3. doi: 10.1111/jdv.15246 30199578

[34] Yu Y Tse K-Y Lee HHY Chow K-L Tsang H-W Wong RWC. Predictive biomarkers and tumor microenvironment in female genital melanomas: a multi-institutional study of 55 cases. Mod Pathol Off J U S Can Acad Pathol Inc. (2020) 33:138–52. doi: 10.1038/s41379-019-0345-2 31383965

[35] Cortellini A Bersanelli M Buti S Gambale E Atzori F Zoratto F. Family history of cancer as surrogate predictor for immunotherapy with anti-pd1/pd-L1 agents: preliminary report of the fami-L1 study. Immunotherapy. (2018) 10:43–655. doi: 10.2217/imt-2017-0167 29562816

